# A Randomized Controlled Trial of Chloroquine for the Treatment of Dengue in Vietnamese Adults

**DOI:** 10.1371/journal.pntd.0000785

**Published:** 2010-08-10

**Authors:** Vianney Tricou, Nguyet Nguyen Minh, Toi Pham Van, Sue J. Lee, Jeremy Farrar, Bridget Wills, Hien Tinh Tran, Cameron P. Simmons

**Affiliations:** 1 Oxford University Clinical Research Unit, Hospital for Tropical Diseases, Ho Chi Minh City, Viet Nam; 2 Hospital for Tropical Diseases, Ho Chi Minh City, Viet Nam; 3 Centre for Clinical Vaccinology and Tropical Medicine, Churchill Hospital, Oxford, United Kingdom; 4 Faculty of Tropical Medicine, Mahidol University, Bangkok, Thailand; Pediatric Dengue Vaccine Initiative, United States of America

## Abstract

**Background:**

There is currently no licensed antiviral drug for treatment of dengue. Chloroquine (CQ) inhibits the replication of dengue virus (DENV) *in vitro*.

**Methods and Findings:**

A double-blind, randomized, placebo-controlled trial of CQ in 307 adults hospitalized for suspected DENV infection was conducted at the Hospital for Tropical Diseases (Ho Chi Minh City, Vietnam) between May 2007 and July 2008. Patients with illness histories of 72 hours or less were randomized to a 3-day course of CQ (n = 153) or placebo (n = 154). Laboratory-confirmation of DENV infection was made in 257 (84%) patients. The primary endpoints were time to resolution of DENV viraemia and time to resolution of DENV NS1 antigenaemia. In patients treated with CQ there was a trend toward a longer duration of DENV viraemia (hazard ratio (HR) = 0.80, 95% CI 0.62–1.05), but we did not find any difference for the time to resolution of NS1 antigenaemia (HR = 1.07, 95% CI 0.76–1.51). Interestingly, CQ was associated with a significant reduction in fever clearance time in the intention-to-treat population (HR = 1.37, 95% CI 1.08–1.74) but not in the per-protocol population. There was also a trend towards a lower incidence of dengue hemorrhagic fever (odds ratio = 0.60, PP 95% CI 0.34–1.04) in patients treated with CQ. Differences in levels of T cell activation or pro- or anti-inflammatory plasma cytokine concentrations between CQ- and placebo-treated patients did not explain the trend towards less dengue hemorrhagic fever in the CQ arm. CQ was associated with significantly more adverse events, primarily vomiting.

**Conclusions:**

CQ does not reduce the durations of viraemia and NS1 antigenaemia in dengue patients. Further trials, with appropriate endpoints, would be required to determine if CQ treatment has any clinical benefit in dengue.

**Trial Registration:**

Current Controlled Trials number ISRCTN38002730.

## Author Summary

There is no available drug or vaccine against dengue, an acute viral disease that affects ∼50 million people annually in tropical and sub-tropical countries. Chloroquine (CQ), a cheap and well-tolerated drug, inhibits the growth of dengue viruses in the laboratory with concentrations achievable in the body. To measure the antiviral efficacy of CQ in dengue, we conducted a study involving 307 adults with suspected dengue. Patients received a 3-day oral dosage of placebo or CQ early in their illness. Unfortunately, we did not see an effect of CQ on the duration of viral infection. We did, however, observe that CQ had a modest anti-fever effect. In patients treated with CQ, we observed a trend towards a lower incidence of dengue hemorrhagic fever, a severe form of dengue. We did not find any differences in the immune response that can explain this trend. We also found more adverse events, primarily vomiting, with CQ. This trial provides valuable new information on how to perform trials of antiviral drugs for dengue.

## Introduction

Dengue is a globally important public health problem. This mosquito-borne viral infection results in an estimated 50 million cases of symptomatic illness each year in over 100 affected countries [1]. There are no licensed vaccines to prevent dengue and no specific therapies to stop or limit viral replication or modulate the severity of symptoms in patients.

Infection with any of the four dengue virus serotypes can cause clinically apparent disease. A measurable viraemia is typically present for the duration of the febrile period, with the first 48–72hrs characterized by relatively high viraemia levels that then rapidly decline as acquired humoral and cellular immune responses resolve infection [2]. NS1, a non-structural protein secreted by virus-infected cells, can be detected in the peripheral blood in some, but not all, symptomatic individuals [3], [4]. Both viraemia and NS1 levels are higher in patients with more severe clinical patterns of disease [5]. The majority of symptomatic infections manifest as an acute systemic febrile illness that is clinically uncomplicated and lasts for 3–7 days. For reasons not fully elucidated, some DENV infections result in severe dengue, a syndrome usually characterized by transiently increased capillary permeability and a hemorrhagic diathesis. Parenteral fluids are used to replenish the intravascular volume and maintain cardiovascular stability during the period of maximum capillary permeability. Mortality in severe dengue can be reduced to less than 1% in experienced settings.

Previous randomized controlled trials in dengue have focused on supportive management and to our knowledge, there has never been a trial directed towards reducing the virus burden. Chloroquine (CQ) is a cheap, widely available and well-tolerated lysosomotropic 4-amino-quinoline derivative. In vitro, CQ has modest anti-viral effects on replication of viruses from diverse taxonomic families (reviewed in [6]). This has led to speculation that CQ could have a therapeutic role in the treatment of viral diseases where there are limited or no other therapies [6], [7]. In the context of DENV, the lysosomotropic and weak base properties of CQ could exert anti-viral activity by interfering with endosomal fusion and furin-dependent virus maturation, which both require low pH environments in late endosomes and the lumen of the trans-Golgi network respectively [8], [9]. Indeed, treatment of mammalian-expressing cells with chloroquine inhibits DENV infection [10]. Furthermore, treatment of DENV-2 infected mammalian cells with chloroquine reduces the infectivity of the produced virus by six- to eightfold, possibly by reducing the efficiency of the virus maturation process [11]. The 50% inhibitory concentration of chloroquine for DENV [10] is achievable inside human cells following ingestion of standard doses of CQ [12], [13]. CQ could also modulate the host response to virus infection. Recognition of viral products by plasmacytoid dendritic cells (pDCs) occurs through a TLR-dependent pathway that requires endosomes acidification [14], [15]; chloroquine-mediated blocking of this process partially inhibited West Nile virus-induced IFN-α production by pDC cultures [16]. CQ could also modulate antigen processing via an increased export of soluble antigens into the cytosol of DCs [17]. CQ also attenuates inflammatory cytokine responses [18], [19] and this may in part explain why CQ is used as a 2^nd^ line therapy in the treatment of inflammatory disorders such as rheumatoid arthritis and systemic lupus erythematosus [20], [21], [22], [23]. Possibly related to its anti-inflammatory properties, CQ exerts an antipyretic effect equal to paracetamol during treatment of uncomplicated *P. falciparum* malaria [24].

Against a backdrop of interest in CQ as a therapeutic for acute viral infections [6], [7], the purpose of this study was to evaluate CQ as potential anti-viral therapy in a randomized, double-blind placebo-controlled trial of adolescents and adults with dengue.

## Materials and Methods

### Study setting, participants and treatment allocation

We performed a randomized (allocation ratio 1∶1), double blind, placebo-controlled parallel-group study in 307 adults hospitalized for suspected DENV infection. Study participants were recruited from the Hospital for Tropical Diseases (HTD) in Ho Chi Minh City, Vietnam. Patients were eligible if they were ≥15 yrs, had a self-reported illness history of 72 hrs or less and were suspected of having dengue. Patients were excluded if they were pregnant or receiving therapy for other chronic disorders, had a history of hypersensitivity to CQ, or written consent from either the patient or a parent was not obtained. Physicians in the Hospital for Tropical Diseases were responsible for enrolment. Patients were randomly assigned to receive CQ (Mekophar Chemical-Pharmaceutical Joint-Stock Company, Ho Chi Minh City, Viet Nam) or placebo. The regimen for CQ was 600mg base (4×150mg tablets) on enrolment to the study, then 600mg on day 2 and 300mg on day 3 (following the World Health Organization recommended treatment regimen for CQ susceptible *P. vivax*) [25]. Patients in the placebo arm received the same regimen of tablets (identical color and size). All treatment courses were contained in identical pre-packed bottles that were randomly assigned to patients via a computer-generated sequence of random numbers in blocks of 20 patients. A pharmacist generated the random sequence and was the only person who knew the content of each bottle. All patients, care providers and study investigators were blinded to treatment assignments. Physicians in the Hospital for Tropical Diseases were responsible for ensuring that the correct sequence of study codes, and therefore the treatment allocation, was followed. The study medication was given within 1 hr of a baseline blood sample being collected. Clinical care, including other treatments such as parenteral fluid therapy was at the discretion of the attending physician and following hospital guidelines. Case classification was according to 1997 WHO classification criteria and was applied to each case after review of study notes [26]. The Scientific and Ethical committee of the HTD and the Oxford Tropical Research Ethical Committee approved the study protocol and all patients gave written informed consent. The trial was registered with the ISRCTN Register (ISRCTN38002730).

### Investigations

#### Clinical and laboratory investigations

Clinical history and examination findings were recorded daily into case record forms. An ultrasound was performed in all patients within 24hrs of defervescence. Venous blood samples were collected at hospital admission (prior to study drug administration), then twice daily (around 9am and 3pm) for a minimum of 5 days after starting treatment (defined as study day 1) and again 10–14 days after discharge from the hospital. Plasma, for use in diagnostic investigations, was stored frozen in multiple aliquots at −80°C until use. A complete blood count, including hematocrit (Hct) and platelet measurements, was performed daily for all patients. Hct measurements were performed more frequently if clinically indicated. The extent of hemoconcentration during symptomatic illness was determined by comparing the maximum Hct recorded during hospitalization with either the value recorded at follow-up when available or against a sex-matched population value.

#### Adverse events

Adverse events (AE) were defined as any unfavorable and unintended abnormal laboratory finding, symptom or disease that occurred during the course of the study, regardless of whether it was considered to be related to the intervention. AEs were classified as mild (grade 1), moderate (grade 2), severe (Grade 3) and life-threatening (grade 4) according to the Common Terminology Criteria for Adverse Events from National Cancer Institute. The relatedness of the AEs to study drug was investigated and graded as definitely, probably, possibly, unlikely to be, or not related.

#### Dengue diagnostics

A diagnosis of “confirmed acute dengue” was reached using previously described serological methods and a diagnostic algorithm [27]. DENV viraemia in plasma was measured using an internally controlled, serotype-specific, real-time RT-PCR TaqMan assay that has been described elsewhere [28]. RNA extraction was automated (NucliSens easyMAG, BioMerieux, Marcy l'Etoile, France). Results were expressed as cDNA equivalents per ml of plasma. Sample measurements were only valid when there was a detectable signal from the internal control amplicon and were considered as positive if above the assay limit of detection defined as the last dilution of standard that gave a specific signal. NS1 was detected by using the NS1 Platelia assay from BioRad (Hercules, CA) according to the manufacturer's instructions.

### Outcome assessment

#### Primary outcomes

The primary endpoints were the time to resolution of viraemia and the time to resolution of NS1 antigenaemia. Time to resolution of viraemia was defined as the time from the start of treatment until the first of two consecutive plasma samples were RT-PCR negative. Time to resolution of NS1 antigenaemia was defined as the time from the start of treatment until the first of two consecutive plasma samples were NS1 ELISA negative. Patients who did not reach viraemia or NS1 antigenaemia clearance were treated as censored at their last date of viraemia or NS1 antigenaemia measurement.

#### Secondary endpoints

The fever clearance time (FCT) was defined as the time from the start of treatment to the start of the first 48 hour period during which axillary temperature remained below 37.5°C. Other pre-defined secondary endpoints were- a) the median nadir platelet count, b) the mean maximum % hemoconcentration (calculated as (maximum hematocrit recorded during the inpatient period)−(hematocrit at follow-up when available or a sex-matched population value)/(hematocrit at follow-up when available or a sex-matched population value)×100), c) the proportion of patients who were treated with intravenous fluid (the decision was based in clinical signs and conducted according to Hospital for Tropical Diseases protocols ; briefly, intravenous fluids were given if the attending physician believed treatment was necessary because of persistent vomiting, gastrointestinal bleeding, hemoconcentration or hypotension), d) the proportion of patients in each arm classified as having dengue hemorrhagic fever (DHF), e) the proportion of patients in each arm with grade 3 or 4 adverse events that were probably or definitely related to the intervention, f) the proportion of patients in each arm with one or more episodes of vomiting and g) the proportion of patients in each arm with bleeding that required blood transfusion.

### T cell and cytokine investigations

To determine the optimal time point for cytokine measurement, levels of IL-1β, IL-6, IL-8, IL-10, IL-12p70, and TNF-α were measured on serial plasma samples from 39 patients by using a CBA Human Inflammatory Cytokines kit (Becton Dickinson, San Jose, CA) according to the manufacturer's instructions (except that all samples were fixed in 4% paraformaldehyde before being analyzed). Subsequently, a luminex-based Bio-Plex system (Bio-Rad Laboratories, Hercules, CA) was used according to the manufacturer's instructions to measure simultaneous plasma levels of IL-2, IL-4, IL-6, IL-8, IL-10, granulocyte macrophage colony stimulating factor (GM-CSF), INF- γ, and TNF-α in 1 plasma sample from each patient. Flow-cytometric analysis of whole-blood samples stained with fluorochrome-conjugated monoclonal antibodies (CD3-Cy, CD4-PE-Cy7, CD8-PE, CD38-FITC, HLA-DR-PerCP and Ki67FITC) was performed by use of a FACScalibur flow cytometer (Becton Dickinson (BD)). Cell-surface staining was routinely performed on 150µL of fresh whole blood. All antibodies were purchased from BD. Whole-blood samples from healthy volunteer subjects were used as group control.

### Sample size calculation

Assuming a median time from enrolment to resolution of viraemia or NS1 antigenaemia in the placebo group of 72 hours and a reduction of this time by 24 hours due to CQ treatment (corresponding to a hazard ratio of 0.67 assuming an exponential distribution of the resolution times), we would need to observe viraemia or NS1 antigenaemia resolution in 191 patients to show such an effect with 80% power at the two-sided 5% significance level. Assuming sufficient follow-up to observe viraemia or NS1 antigenaemia resolution in 90% of patients, we would need to include at least 213 patients with confirmed dengue.

### Statistical methods

The statistician was unblinded for the data analysis. Data stayed blinded until the database was cleaned and locked ready for data analysis. All statistical analyses were performed using Intercooled STATA version 9.2 (StataCorp, TX). A two-sided p-value ≤0.05 was considered significant for all parameters. The intention-to-treat (ITT) population was defined as all subjects who were randomized regardless of whether or not they began the treatment regimen. All laboratory confirmed dengue patients completing the expected number of days of treatment who fulfilled the inclusion/exclusion criteria of the protocol and who did not leave before the end of the study drug course formed the per-protocol (PP) population. Secondary endpoints (except the FCT) were compared between the 2 groups and analyzed using the Kruskal-Wallis test for continuous variables and the Fisher's exact test for categorical variables. For the primary endpoints and the FCT, the null hypothesis is that CQ has no effect on duration of DENV viraemia, NS1 antigenaemia and fever. Survival analysis using the Kaplan-Meier (KM) method and log-rank test was used for all time-to-event outcomes. Cox regression was used to quantify the difference in risk between treatment groups and to adjust for all the following baseline variables: time since illness onset at enrolment, serological status, serotype (DENV-1 vs other), viraemia and temperature. Because these covariates were thought to influence the time to resolution of viraemia, the time to resolution of NS1 antigenaemia and the FCT, all were retained in the final adjusted models. The proportional hazards assumptions were checked using a test based on Schoenfeld residuals.

## Results

### Baseline characteristics of enrolled patients

**Figure 1:**
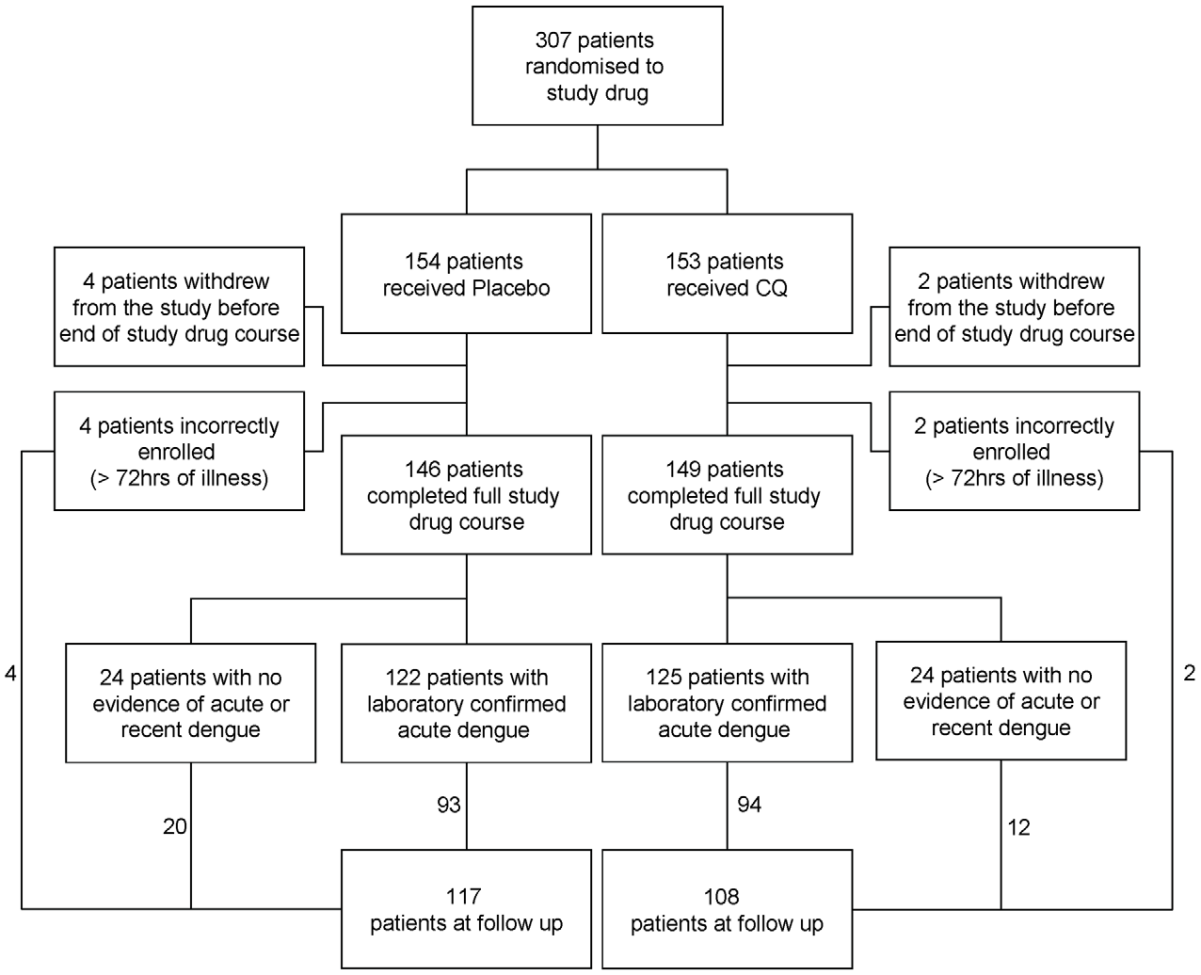
Participant flow in the randomized controlled trial of CQ vs. Placebo.

Between May 2007 and July 2008, 307 adults with suspected dengue were randomized to CQ or placebo (Fig. 1). Of these 307 patients, 257 had laboratory confirmed dengue and 50 had no evidence of recent or acute dengue. All patients recovered fully. The baseline characteristics of the study population are summarized in Table 1. Baseline characteristics were generally well-balanced between the two groups except for baseline viraemia which tended to be higher in the CQ group (median 9.04 vs 8.52 Log10 copies/mL) and the proportion of DENV3 infected patients, which was lower in the CQ arm (11.3% CQ vs 21.8% placebo).

**Table 1:** Baseline characteristics in the intention-to-treat population.

Variables	CQ group (N = 153)	Placebo group (N = 154)
	N (%) or Median (interquartile range)	
**Age (years)**	22 (18–27)	22 (19–28)
**Male sex**	104 (68.0%)	106 (68.8%)
**Dengue confirmed**	128 (83.7%)	129 (84.3%)
**Viremic**	124 (81.0%)	124 (80.6%)
**Infecting serotype:**		
**DENV-1**	80 (64.5%)	67 (54.0%)
**DENV-2**	26 (21.0%)	27 (21.8%)
**DENV-3**	14 (11.3%)	27 (21.8%)
**DENV-4**	4 (3.2%)	3 (2.4%)
**viraemia (log10 copies/mL of plasma):**		
**All serotypes**	9.0 (8.0–9.6)^a^	8.5 (7.6–9.3)
**DENV-1**	9.2 (8.3–9.8)	8.9 (7.9–9.7)
**DENV-2**	8.3 (7.0–9.4)^a^	7.7 (6.3–8.6)
**DENV-3**	8.3 (7.6–9.1)	8.2 (7.4–9.3)
**DENV-4**	7.7 (6.9–8.7)	7.7 (6.0–8.5)
**Serological status:**		
**Primary**	20 (15.7%)	12 (9.3%)
**Secondary**	105 (82.7%)	110 (85.3%)
**Ambiguous**	2 (1.6%)	7 (5.4%)
**NS1 ELISA positive**	113 (73.9%)^b^	110 (71.4%)
**Febrile**	150 (98.0%)^c^	147 (95.5%)^c^
**Temperature (°C)**	39.3 (38.7–40)	39.5 (38.8–40)
**Time since illness onset (hrs)**	49 (40–56)	48 (40–57)
**Platelet count at enrolment**	129,000 (95,000–168,000)	125,000 (91,000–167,000)

a Baseline viraemia value missing for 1 patient in the CQ arm.

b Two patients NS1 negative at enrolment became positive later (both in the CQ arm).

c Three patients afebrile at enrolment later developed fever (1 in the CQ arm and 2 in the Placebo arm).

### Primary endpoints

#### DENV viraemia clearance times

**Figure 2:**
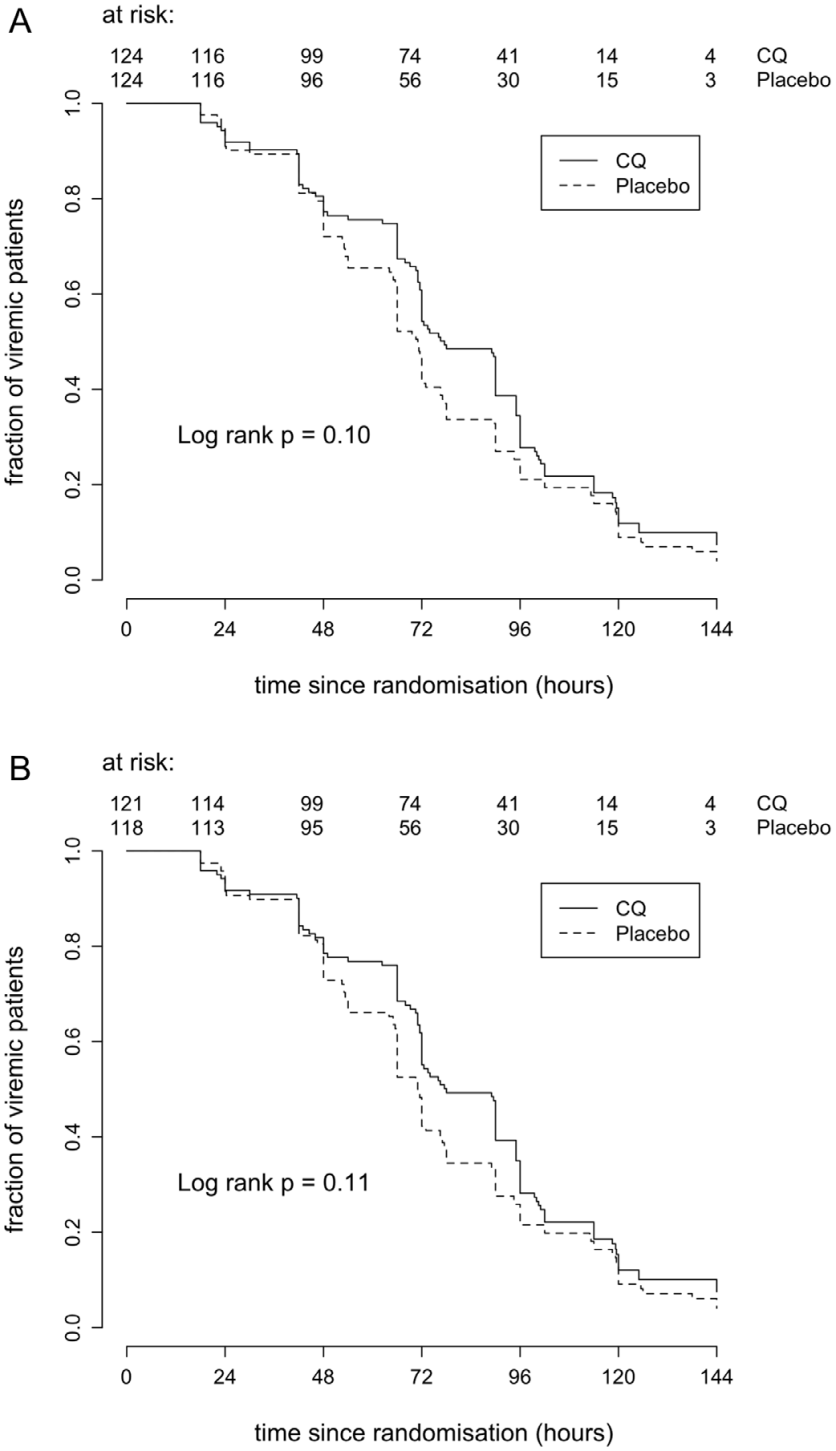
Time to fever clearance. Kaplan – Meier survival analysis of time to fever clearance by treatment group (CQ or placebo) and population; **A**) Intention to treat population and **B**) Per Protocol population. Three patients afebrile at enrollment developed fever later (1 in the CQ arm and 2 in the Placebo arm) and for the purposes of analysis were considered positive at the time of enrolment.

There were 248 patients viraemic at enrolment in the ITT population and 239 in the PP population (Table 1). Viraemia clearance times were not significantly different in the CQ arm compared to the placebo arm for either the ITT or PP analysis (Fig. 2A and B) (ITT hazard ratio (HR) 0.80, 95% CI 0.62–1.05, log rank test P = 0.10 and PP HR = 0.80, 95% CI 0.61–1.05, log rank test P = 0.11). Median times to resolution of DENV viraemia were similar in the ITT and PP population: ITT 77.5hrs and PP 78hrs (ITT inter-quartile range (IQR) 53–100hrs and PP IQR 66–100.5hrs) for CQ arm and both ITT and PP 71hrs (IQR ITT 48–94.5hrs and PP 48–95.5hrs) for Placebo arm. Adjusting for baseline covariates did not alter these findings (ITT HR = 0.95, 95% CI 0.72–1.26 and PP HR = 0.94, 95% CI 0.71–1.25). Of the 24 patients still qRT-PCR positive at discharge (median discharge time for those patients = 5 days since enrolment) only 14 presented at follow-up and none were qRT-PCR positive (median follow up time for those patients = 13 days after enrolment).

#### Time to negative NS1 antigenaemia

**Figure 3:**
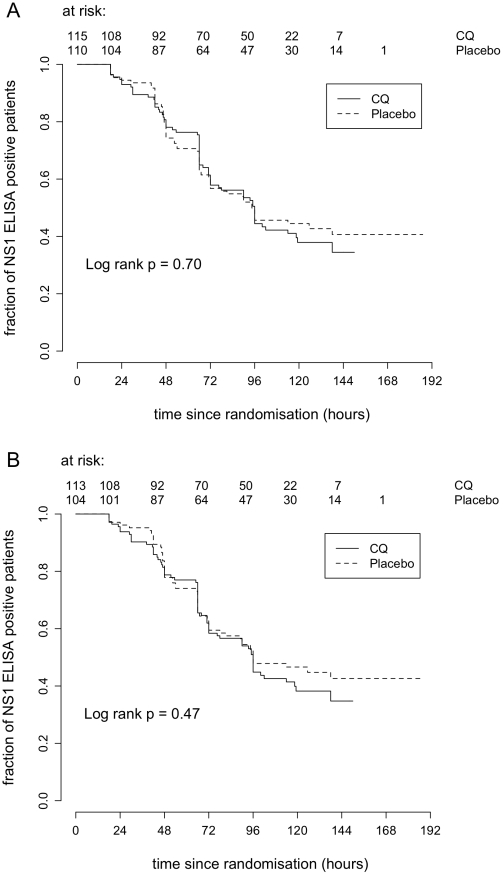
Time to resolution of viraemia. Kaplan – Meier survival analysis of time to resolution of plasma viraemia by treatment group (CQ or placebo) and population; **A**) Intention to treat population and **B**) Per Protocol population.

In the ITT population, there were 223 (72.6%) patients NS1 positive at the time of enrolment (plus 2 patients negative at enrolment but NS1 positive 24 and 42hrs later) (Table 1). Time to resolution of NS1 antigenaemia was not significantly different between CQ and placebo arms (Fig. 3A) (HR = 1.07, 95% CI 0.76–1.51, log rank test *P* = 0.70). Adjusting for baseline covariates did not alter these findings (HR = 1.18, 95% CI 0.82–1.68). Median times to resolution of NS1 antigenaemia were 96hrs (IQR 65.5–115hrs) in the CQ arm and 94.5hrs (48–120hrs) in the placebo arm. There were 96 patients still NS1 ELISA positive at discharge. This suggests that NS1 antigenaemia is relatively long lived. Moreover 17 patients (∼27% of patients NS1 positive at discharge and with a follow-up sample) were still NS1 positive at follow-up (time range: 10.7–14 days after enrolment).

In the PP population, there were 215 (87.0%) patients NS1 positive at the time of enrolment (plus 2 patients negative at enrolment but NS1 positive 24 and 42hrs later) (Table 1). Time to resolution of NS1 antigenaemia was not significantly different between CQ and placebo arms (Fig. 3B) (HR = 1.14, 95% CI 0.80–1.63, log rank test *P* = 0.47). Adjusting for baseline covariates did not alter these findings (HR = 1.19, 95% CI 0.83–1.71). Median times to resolution of NS1 antigenaemia were 96hrs (IQR 66–116hrs) and 96hrs (54–120hrs) respectively for CQ and placebo arms.

### Secondary endpoints

#### Fever clearance times

**Figure 4:**
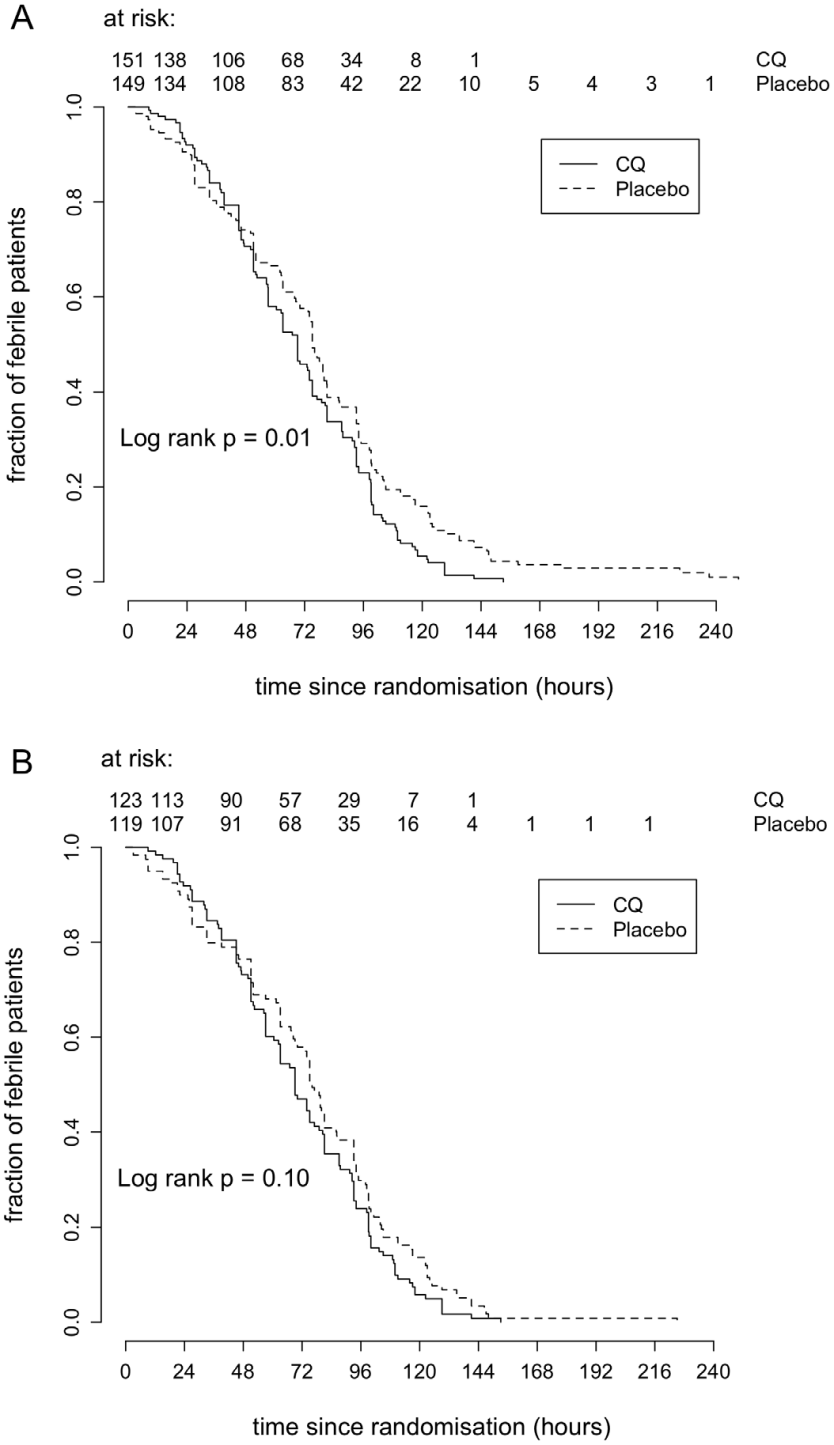
Time to negative NS1 antigenaemia. Kaplan – Meier survival analysis of time to resolution of NS1 antigenaemia by treatment group (CQ or placebo) and population; **A**) Intention to treat population and **B**) Per Protocol population. Two patients NS1 negative at enrollment were later positive (both in the CQ arm) and for the purposes of analysis were considered positive at the time of enrolment.

In the ITT population, there were 297 patients febrile at enrolment (plus 3 afebrile who developed fever soon after) (Table 1). FCTs were significantly shorter in the CQ arm compared to the placebo arm (HR = 1.37, 95% CI 1.08–1.74, log rank test P = 0.01 but there was a trend that the hazards were non-proportional p = 0.07) (Fig. 4A). However, when adjusted for baseline covariates, the rate of fever clearance among patients who received CQ was not different from patients who received placebo (HR = 1.16, 95% CI 0.89–1.51, P = 0.28). Median FCTs were 69hrs (IQR 45–93hrs) and 75hrs (IQR 36.5–99hrs) respectively for CQ and placebo arms.

There were 240 patients febrile at enrolment (plus 2 afebrile who developed fever soon after) in the PP population (Table 1). FCTs were not different between the 2 groups (HR = 1.24, 95% CI 0.96–1.60, log rank test P = 0.10) (Fig. 4B). Adjusting for baseline covariates did not alter these findings (HR = 1.28, 95% CI 0.98–1.68, P = 0.07). Median FCTs were 69hrs (IQR 45–93hrs) and 76hrs (IQR 46–99hrs) respectively for CQ and placebo arms.

#### Platelet nadir and maximum hemoconcentration

The median nadir platelet count was the same in the CQ and placebo arms 45,000 (IQR 25,000–60,000) (Mann-Whitney p = 0.61, PP population). There were also no significant differences between the two arms in the mean level of hemoconcentration detected (10.8% (8.9–12.7) and 12.2% (10.3–14.1) for the CQ and placebo respectively (Mann-Whitney p = 0.27, per protocol).

#### DF vs. DHF in each arm

There was a trend, though not significant (P = 0.09), towards fewer patients with DHF in the CQ arm (Table 2). There were 29 patients (23.2%) with DHF in the CQ arm compared to 41 (33.6%) in the Placebo arm (odds ratio 0.60, 95% CI 0.34–1.04, P = 0.07). As the infecting DENV serotype might influence clinical severity, and at baseline the two arms differed in the prevalence of each serotype, the analysis of the effect of CQ on disease severity was also adjusted for serotype by logistic regression, but this did not alter these findings.

**Table 2:** Summary of secondary endpoints.

	CQ N (%)	Placebo N (%)	P value^a^
**Intention to treat population**	**153**	**154**	
Patient with AE	18 (11.8%)	6 (3.9%)	0.01
Patient with vomiting	15 (9.8%)	6 (3.9%)	0.04
Patient requiring IV fluid	21 (13.7%)	11 (7.1%)	0.06
**Per protocol population**	**125**	**122**	
Patient with AE	17 (13.6%)	5 (4.1%)	0.01
Patient with vomiting	14 (11.2%)	5 (4.1%)	0.05
Patient requiring IV fluid	19 (15.2%)	10 (8.2%)	0.11
DHF patient	29 (23.2%)	41 (33.6%)	0.09

a Fisher's exact test.

#### Adverse events

Two patients in the CQ arm developed severe adverse events (both grade 3) that were possibly related to CQ. One patient with hematemesis was admitted to the ICU for 3 days, with stable vital signs. The 2^nd^ patient was anorexic and vomiting with a narrow pulse pressure 100/80 mmHg) and was admitted to the ICU for 3 days. There were no severe AEs in the placebo arm. Significantly more adverse events occurred in the CQ arm: 18 patients reported a total of 33 AEs versus 6 patients with 8 AEs in the placebo arm (Fisher's exact test P = 0.01) (Table 2). The most common adverse event was vomiting (∼51% of all grade adverse events) (Table 2).

### Number of patients requiring fluid and blood transfusion

Thirty two patients (21 in CQ arm and 11 in placebo arm) required parenteral crystalloid fluid therapy during their hospitalization (for rehydration, and/or maintenance) but none required blood transfusion (Table 2). There was no significant difference between the 2 groups in the need for fluid therapy (p-value = 0.11 in the PP population and 0.06 in the ITT population).

### T cell activation in peripheral blood of study participants

**Figure 5:**
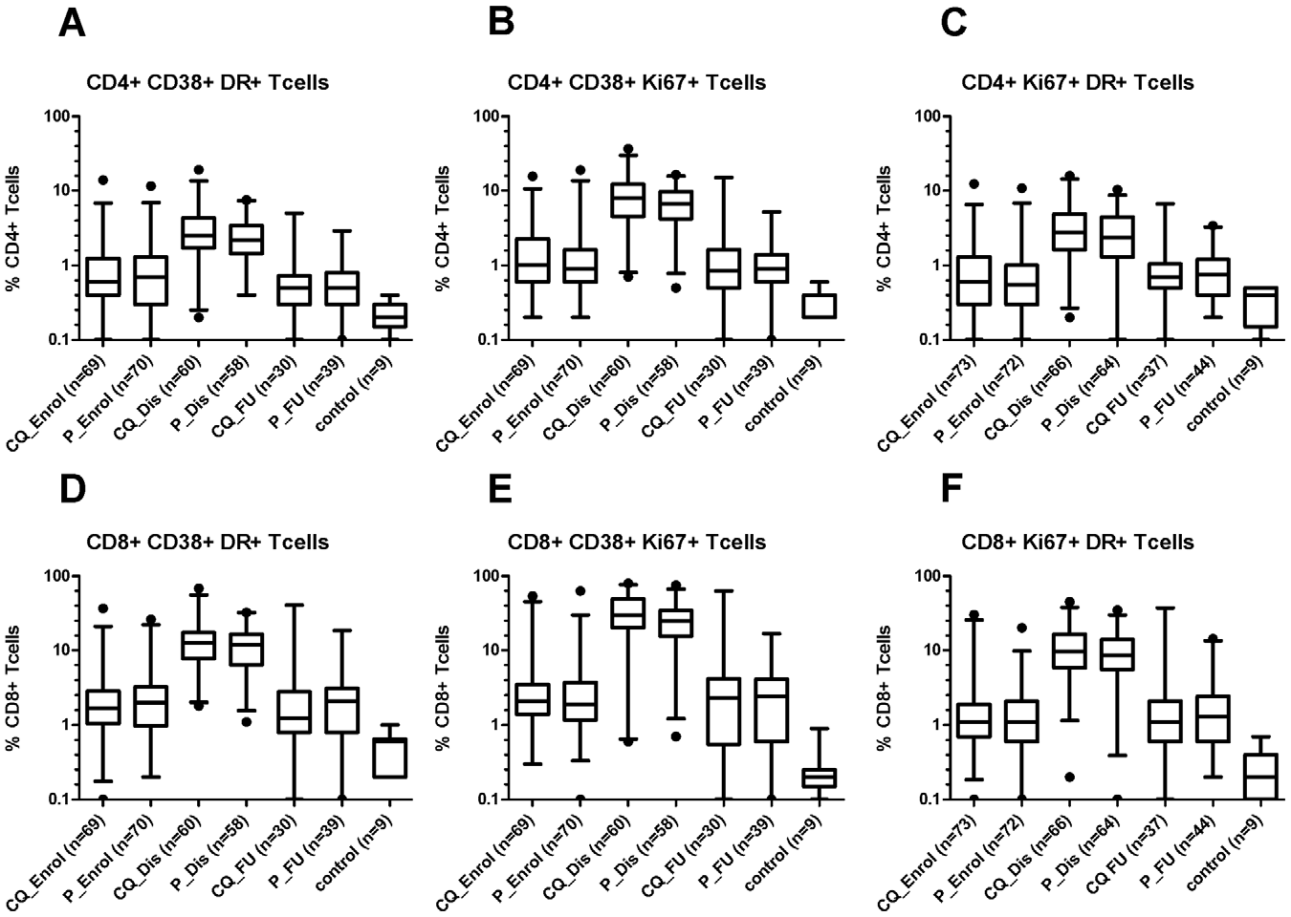
Surface phenotypes of CD4^+^ and CD8^+^ T cells in laboratory-confirmed dengue patients randomized to placebo or CQ. The Box and Whisker plots show the median number and range (2.5–97.5 percentile) of percentages of surface-activated T cells in peripheral blood from CQ (n = 74) and Placebo (n = 73) treated laboratory-confirmed dengue patients at different time points. The median illness day (range) for enrolment samples was 2 (0–3) days, for hospital discharge was 6 (4–8) days and for follow-up was 15.5 (13–30) days. Shown are percentages of peripheral blood CD4^+^ T cells that were **A**) CD38^+^HLA-DR^+^, **B**) CD38^+^Ki67^+^, and **C**) Ki67^+^ HLA-DR^+^. Also shown are percentages of CD8^+^ T cells that were, **D**) CD38^+^, HLA-DR^+^, **E**) CD38^+^Ki67^+^, and **F**) Ki67^+^ HLA-DR^+^. The labels below the graphs indicate the time at which sample collection occurred.

Given the clinical experience of using CQ therapy in inflammatory autoimmune disorders, we investigated whether CQ was associated with a measurable attenuation of the T cell response. To this end, the activation state of peripheral blood CD3^+^CD4^+^ and CD3^+^CD8^+^ T cells was assessed in fresh whole-blood at the time of enrolment, on illness day 6, and again at follow-up in 172 consecutive patients enrolled in the study between September 07 and June 08 (85 in CQ arm, 87 in placebo arm), amongst whom there were 147 laboratory-confirmed dengue patients. The activation markers used were CD38, HLA-DR and Ki-67. As a reference, we also phenotyped T cells in fresh whole blood from 9 healthy adult volunteers. Strikingly, in dengue patients we observed a large population of surface-activated (CD38^+^ or HLA-DR^+^) and proliferating (Ki-67^+^) CD8^+^ T cells at early convalescence that were mostly absent at the time of enrolment and follow-up (Fig. 5). There was no evidence however of a significant difference in the proportion of activated T cells in patients treated with CQ or placebo.

### Plasma concentrations of cytokines/chemokines

**Figure 6:**
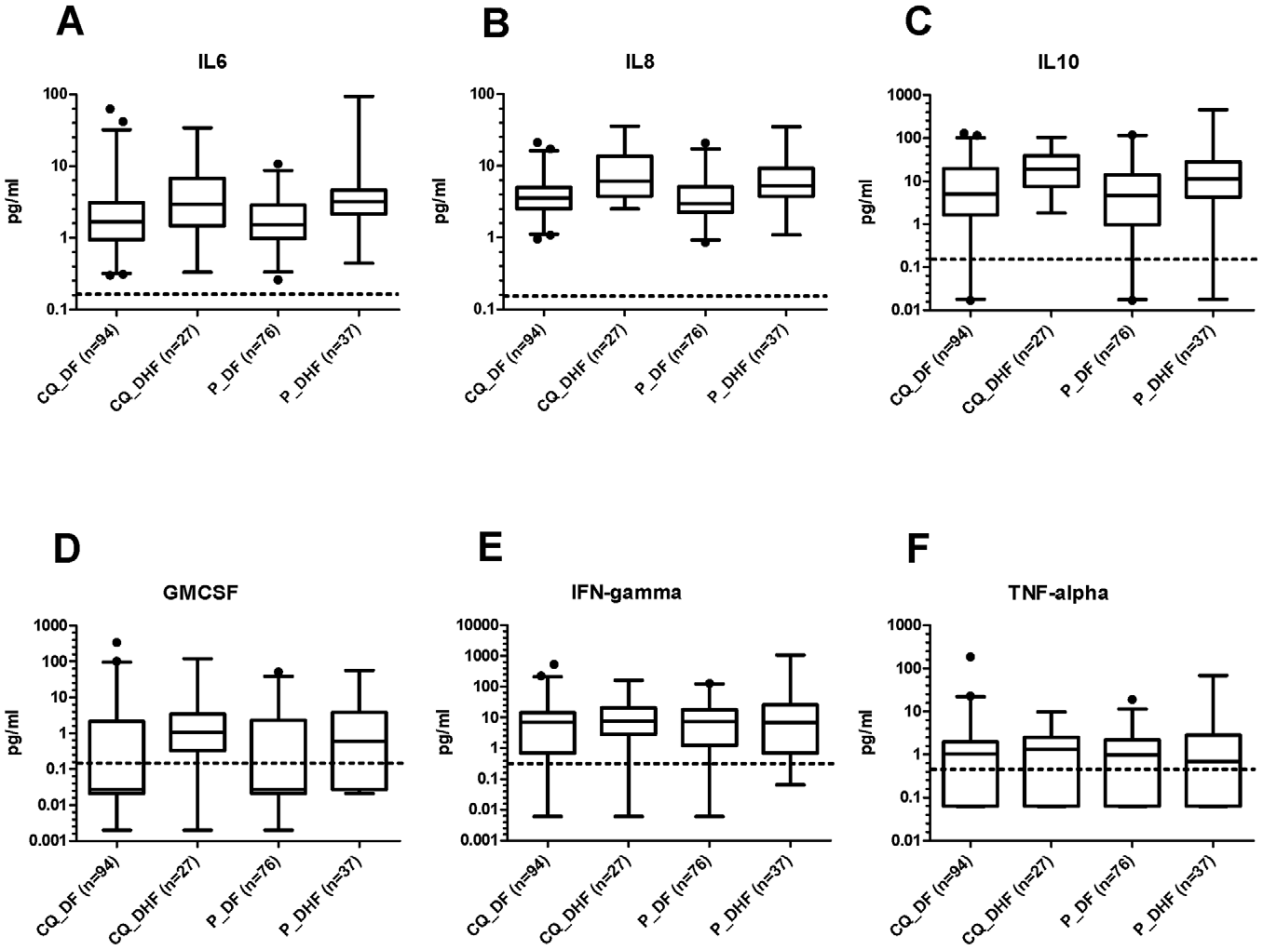
Plasma concentrations of pro- and anti-inflammatory cytokines in laboratory-confirmed dengue patients randomized to placebo or CQ. The Box and Whisker plots show the median and range (2.5^th^–97.5^th^ percentile) of plasma concentrations of **A**) IL-6, **B**) IL-8, **C**) IL-10, **D**) GM-CSF, **E**) IFN- γ, and **F**) TNF-α from DF and DHF patients treated with CQ or Placebo. The dashed line represents the assay limit of detection. Concentrations of IL-2 and IL-4 are not shown because they were below the limit of detection.

To understand if CQ modulated the cytokine response to DENV infection, plasma concentrations of IL-2, IL-4, IL-6, IL-8, IL-10, GM-CSF, INF-γ, and TNF-α were measured in plasma from 234 laboratory-confirmed dengue patients (121 in CQ arm, 113 in placebo arm) 2 or 3 days after randomization (Fig. 6). However, there was no significant difference in plasma concentrations of any of these cytokines between CQ or placebo treated patients (Mann-Whitney P>0.1).

## Discussion

There are no specific therapies for treating dengue. This controlled trial was conducted to determine if CQ could reduce the viral burden in dengue patients. We found no evidence that CQ reduced the duration of viraemia or NS1 antigenaemia in adult dengue patients, but did observe a modest anti-pyretic activity of CQ in the intention to treat population, but not in dengue laboratory-confirmed cases. CQ was associated with a higher frequency of adverse events compared to placebo, but these were generally mild. There was no evidence that CQ reduced the magnitude of cytokine or T cell responses to DENV infection.

To our knowledge the only previous therapeutic trial of CQ for an acute viral infection has been in a small number of patients with Chikungunya virus infection [29], in which CQ had no impact on either duration of febrile arthralgia or viraemia. Several possible reasons could explain the lack of measurable activity in this study of CQ against virological markers of DENV infection in vivo. Although the C_max_ of CQ inside cells approximates the IC_50_ value of CQ against DENV in vitro, it is possible that CQ does not achieve inhibitory concentrations inside the reticuloendothelial cells where DENV replication is believed to occur [30]. Furthermore, it may not achieve the same pH modulation in vivo that is postulated to explain its activity on cultured virus in vitro.

Alternative trial designs and protocols, such as increasing the therapeutic dose, dosing patients earlier in their illness or increasing the sample size substantially might increase the chances of observing an in vivo effect by CQ on the duration of DENV viraemia and NS1 antigenaemia. The importance of treating early is highlighted by the fact that in this trial the median duration of illness prior to treatment was relatively short (∼48 hrs) and the median viraemia clearance times after treatment were ∼3.75 days in the CQ arm and ∼3 days in the placebo arm. Strikingly however, the duration of NS1 antigenaemia was relatively long, with as many as 92/243 (38%) of dengue patients still NS1 positive at the time of discharge from hospital, although most of this antigen is probably generated in the first few days of illness and its prolonged clearance simply reflects its large, oligomeric structure [31], [32]. The time to resolution of NS1 antigenaemia may therefore not be an optimal endpoint and an alternative approach could have been to compare the proportion of patients that were positive at a single post-therapy timepoint (e.g. study day 5). Collectively, these data underscore that there is only a brief therapeutic window of opportunity to improve upon the host's virus-eliminating immune response. Encouragingly however, strategies to diagnose patients very early in their illness are available in the form of NS1 rapid diagnostic tests [27], [33], [34], [35] and these could in principal guide rational treatment with an anti-viral or other intervention as early as 24–48hrs into the illness course. Of additional value, but not yet identified, would be early prognostic markers of severe outcome, so that interventions can be delivered to those patients at higher risk.

A CQ-mediated anti-pyretic effect equal to paracetamol has been shown during treatment of uncomplicated *P. falciparum* malaria [24], [36], [37]. This effect may be explained by CQ's anti-inflammatory properties, including CQ effects on TLR signaling [38], [39]. Fever during an infection is thought to be initiated by virtually immediate cyclooxygenase-2, prostaglandin E2 (PGE2) production, activation of hypothalamic PGE2 receptors and then cytokines and TLR ligand activity [40]. It is reasonable to believe that CQ mediates an anti-pyretic effect by altering the levels and balance of these pyretic mediators during infection. Accordingly, we found a small reduction in fever clearance median times (∼6 hrs) amongst CQ patients in the intention-to-treat patient population, and whilst a similar trend was observed amongst the dengue confirmed patients, it was not statistically significant. CQ might be a better anti-pyretic in non-dengue patients in this study because these patients had milder infections, albeit of unknown origin.

Fewer patients receiving CQ developed DHF. The intriguing possibility that CQ mediated an anti-disease effect, but not a measurable anti-viral effect in this trial is plausible given the literature on CQ as a pleotropic immune-modulatory drug. To find support for this possibility we measured pro- and anti-inflammatory plasma cytokine concentrations and T cell activation markers in dengue patients. Of particular interest were vasodilatory and pyretic cytokines such as TNF-α that have been identified as susceptible to CQ modulation [18], [41] and important in the pathogenesis of the dengue capillary leak syndrome [42]. Similarly, the magnitude of T cell activation has been postulated to be associated with dengue severity [43]. Whilst robust T cell activation and cellular proliferation was indeed present around the time of defervescence, there was no evidence of a difference between CQ and placebo arms for the cellular markers we investigated nor in the cytokines that were measured. The absence of a measurable impact by CQ on these elements of the host response might suggest any trend towards less DHF in the CQ arm is simply chance or reflects our inability to identify and measure true immunological correlates of disease. Only further large trials, with clinical endpoints, will determine if CQ has a disease modulating effect.

Our study had several limitations. The study was hospital-based and therefore the patient population, although presenting early in their illness, may not reflect that seen in primary health care settings where milder infections might be expected. The study was performed in adults, who generally compensate well for capillary permeability, and it's plausible that different findings might be observed in children, who in most endemic settings carry much of the disease burden. We measured viraemia by quantitative RT-PCR as a surrogate and well characterised marker of infection though we recognise this is not that same as a quantitative biological assay of infectious virus. Finally, we did not formally conduct pharmacokinetic analysis of CQ in treated patients and this could have aided the interpretation of the final outcomes.

There is growing interest in the potential for anti-viral therapies for dengue [44], [45]. This study illuminated several important issues in the design of anti-viral interventions trials. Most striking is the rapid decline in the DENV viraemia beginning ∼72hrs into the illness, highlighting the fact that anti-viral interventions will likely need to be delivered very early and aggressively, preferably guided by cheap, sensitive and specific diagnostics. NS1 is a useful and easily assayed biomarker of DENV infection and in the context of a trial it conceivably provides a slightly different insight into virus infection than is given by measurement of viral RNA in plasma. In early phase trials, measurement of virological and clinical markers at multiple time-points per day is strongly recommended given the speed of viral clearance and evolution of disease. In later phase trials, the choice of clinical endpoints will depend on the target patient population and the setting. In children, single or combination endpoints around dengue shock syndrome, the most common life-threatening complication in children, should be considered. In adults, other complications such as severe bleeding may also be relevant.

In summary, this study suggests CQ has no measurable impact on virological or immunological parameters of DENV infection in young adults. We also found no convincing evidence that CQ reduces the time to fever resolution in adults with dengue. Interventions with either more potent anti-viral molecules and/or immunomodulatory drugs are needed to improve clinical outcomes for patients in endemic settings.

## Supporting Information

Checklist S1 CONSORT Checklist
(0.18 MB DOC)

Protocol S1 Trial Protocol
(0.14 MB DOC)
