# Correction: A Randomized Controlled Trial of Chloroquine for the Treatment of Dengue in Vietnamese Adults

**DOI:** 10.1371/annotation/c5c14905-8792-4d2e-8179-f8c70064e773

**Published:** 2012-06-22

**Authors:** Vianney Tricou, Nguyet Nguyen Minh, Toi Pham Van, Sue J. Lee, Jeremy Farrar, Bridget Wills, Hien Tinh Tran, Cameron P. Simmons

The correct title and legend of Figure 4 presently appears with Figure 2. The correct, complete Figure 4 legend and title are:

Time to fever clearance.

Kaplan – Meier survival analysis of time to fever clearance by treatment group (CQ or placebo) and population; A) Intention to treat population and B) Per Protocol population. Three patients afebrile at enrollment developed fever later (1 in the CQ arm and 2 in the Placebo arm) and for the purposes of analysis were considered positive at the time of enrolment.

doi:10.1371/journal.pntd.0000785.g002 

