# Correction: A Randomized Controlled Trial of Chloroquine for the Treatment of Dengue in Vietnamese Adults

**DOI:** 10.1371/annotation/e00ee8fb-4ab9-46db-be8e-3696bb362db4

**Published:** 2012-06-22

**Authors:** Vianney Tricou, Nguyet Nguyen Minh, Toi Pham Van, Sue J. Lee, Jeremy Farrar, Bridget Wills, Hien Tinh Tran, Cameron P. Simmons

The correct title and legend of Figure 3 presently appears with Figure 4. The correct, complete Figure 3 legend and title are:

Time to negative NS1 antigenaemia.

Kaplan – Meier survival analysis of time to resolution of NS1 antigenaemia by treatment group (CQ or placebo) and population; A) Intention to treat population and B) Per Protocol population. Two patients NS1 negative at enrollment were later positive (both in the CQ arm) and for the purposes of analysis were considered positive at the time of enrolment.

doi:10.1371/journal.pntd.0000785.g004

