# Correction: A Randomized Controlled Trial of Chloroquine for the Treatment of Dengue in Vietnamese Adults

**DOI:** 10.1371/annotation/8683caec-b309-46d7-bc47-dc9cc27108e4

**Published:** 2012-06-22

**Authors:** Vianney Tricou, Nguyet Nguyen Minh, Toi Pham Van, Sue J. Lee, Jeremy Farrar, Bridget Wills, Hien Tinh Tran, Cameron P. Simmons

The correct title and legend of Figure 2 presently appears with Figure 3. The correct, complete Figure 2 legend and title are:

Time to resolution of viraemia.

Kaplan – Meier survival analysis of time to resolution of plasma viraemia by treatment group (CQ or placebo) and population; A) Intention to treat population and B) Per Protocol population.

doi:10.1371/journal.pntd.0000785.g003

