# Supplementary material for: A Randomized Controlled Trial of Chloroquine for the Treatment of Dengue in Vietnamese Adults
Source: PLoS Negl Trop Dis. 2010 Aug 10;4(8):e785. doi: 10.1371/journal.pntd.0000785 (PMC2919376; doi:10.1371/journal.pntd.0000785)
Supplement: Protocol S1 — Trial Protocol (0.14 MB DOC) [file pntd.0000785.s002.doc]

**Chloroquine for dengue.**

**Study Code: FR**

**OXTREC 005-06**

**1. Principal investigators**

Dr Tran Tinh Hien, Dr Cameron Simmons, Dr Jeremy Farrar

**2. Institution and Department**

Oxford University Clinical Research Unit Ho Chi Minh City Viet Nam

**3. Project Title**

A double-blind, randomized, placebo-controlled, trial designed to assess the antiviral efficacy of Chloroquine in adult patients hospitalized with dengue.

**Proposed start date:** May 2007.

**4. Data and Safety Monitoring Committee**

Dr Phuong Quoc Tuan (OUCRU), Professor Nick White (Wellcome Trust/Bangkok), Dr Guy Thwaites (Imperial College, UK), Dr Paul Young (University of Queensland, Australia)

**Introduction and rationale**

Dengue fever (DF) is the most common vector-borne viral disease of humans, with more than 100 million cases recorded each year in tropical and subtropical countries and an increasing incidence in more temperate regions. Over half of the world’s population live in areas at risk of infection. In its most serious forms, dengue haemorrhagic fever (DHF) and dengue shock syndrome (DSS), it is also a major cause of morbidity and mortality, particularly in Southeast Asia where it is the principle reason for paediatric admission to hospital during the rainy season. In VietNam over the past three years we have noted an increasing incidence in adults. Data from dengue protein crystallographic studies have revealed opportunities to intervene in the replication cycle of dengue viruses (DENV). The pH-dependent structural changes involving the fusion loop in the envelope protein are central to successful virion fusion with the endosomal membrane and release of infectious viral nucleic acid into the cytoplasm (1). Chloroquine, a safe and pharmacologically well-characterized drug, inhibits several viruses requiring a pH dependent step for cell entry (2)(3). The in vitro inhibitory concentration (IC_50_) for chloroquine against dengue viruses is ~5 μM (Fig. 1 from Novartis Institute for Tropical Diseases, Singapore). Furthermore, chloroquine inhibits the normal proteolytic processing of certain dengue virus proteins thereby reducing the infectivity of the mature virions (4). Following a 3-day course of therapeutic oral doses of chloroquine (600mg base on days 1 and 2, then 300mg base on day), the peak drug concentration (Cmax) ranges from 0.1 to 0.3μg/mL (~0.3-1μM) in plasma and 1 to 1.2μg/mL (3.2-3.8μM) in whole blood. Chloroquine is extensively sequestered in the tissues. In animals, from 200 to 600 times the plasma concentration may be found in the liver, spleen, kidney, and lung (5, 6). In humans, the highest concentrations of approximately 100μM were measured in the marrow, liver, spleen and leukocytes (7). These data suggest a therapeutic potential for chloroquine in dengue-infected patients since the Cmax in relevant organs is likely to exceed the IC50 value. This trial asks the question whether chloroquine, by its action on the acidification of the intracellular vacuole, reduces viral load in patients with dengue.

**Study Aims**

To measure the antiviral efficacy of chloroquine in the treatment of dengue.

Primary endpoint

The primary endpoint is;

1. time to negative DENV NS1 antigenaemia in plasma
2. time to negative reverse transcriptase (RT)-PCR detection of viral RNA in plasma

For the purposes of the study, time to a negative NS1 or PCR will be the interval between starting treatment and the first time NS1 or PCR is negative and remains negative for two consecutive measurements.

#### Sample Size

The following assumptions were made in calculating the sample size for EF study:

1. The median time to NS1 and PCR clearance in dengue population with normal standard of care is 72 hours
2. Most (90%) of dengue patients admitted to the trial will be day 3 of illness
3. The average proportion of cases that are NS1 and/or PCR positive on day 3 is 80%
4. At the end of follow-up (5 days after study entry), 20% of cases will still be positive for NS1
5. 10% of patients lost to follow-up

Under these assumptions, the minimum number of NS1 or PCR clearance cases required to be able to detect a 36 hours reduction of median time in Chloroquine arm as compared to standard care arm with a power of 0.8 and type I error 0.05--equivalently a hazard ratio relating to NS1 clearance of 2—is 72. Therefore total number will be 138/{0.8*(1-0.1)*(1-0.1)} =220 or 110 patients in each arm. The effect size in this sample size calculation study is large, but can be justified on the basis that a smaller effect size is unlikely to be clinically significant.

**Patient selection:**

Patients will be eligible to be admitted to the study if they have suspected uncomplicated dengue and:

1. Are >14 years of age
2. They have a history of symptoms of 3 days or less.
3. They give fully informed consent.
4. No previous history of hypersensitivity to Chloroquine
5. They are not pregnant.
6. They are not G6PD deficient
7. They are not receiving therapy for other disorders

#### Randomisation and Treatment

This will be a double-blind randomised comparison. The randomization code will be computer generated and will be made for blocks of 40 cases. The study drugs will be presented in sealed plastic bottles; each bottle will contain sufficient tablets to complete one course of treatment (10 tablets, 150mg chloroquine base per tablet). Once the bottle is opened the patient will be considered to have entered the trial whatever happens subsequently. Patients will be randomized to group A or group B.

Group A: Chloroquine: 600mg base (4 tablets) on admission to the study, then 600mg 24hrs later (day 2), then 300mg on day 3.

GroupB: Placebo. Same regime of tablets (identically sized) as per chloroquine arm

#### Observations and Investigations

A full history and clinical examination will be performed at study entry and every day during the inpatient period. All clinical data will be documented in the case record form. The following investigations will be performed;

1. Chest X-ray or ultrasound on day of defervescance.
2. Hct, white cell count/differential, platelet count, SGOT, SGPT daily
3. Twice daily viral culture from plasma for minimum 5 days
4. Twice daily quantitative viral PCR from plasma for minimum 5 days
5. Twice daily assessment of Dengue NS1 levels for minimum 5 days
6. Twice daily measurement of plasma concentrations of the pro-inflammatory cytokines TNF, IL-6, IL-1B, IL12, IL-8 and the anti-inflammatory, IL-10 for minimum 5 days
7. ELISPOT assays to quantitate DENV-specific T cell responses
8. Microarray and PCR investigations to measure the effect of chloroquine on host gene-expression responses during acute disease

**Trial monitoring and Adverse events**

There will not be an interim analysis of the trial because of the relatively small sample size. All serious adverse events will be reported to the independent data safety and monitoring committee (DSMC). All adverse events will be recorded in the adverse events form found in the case record forms.

**Blood sample collection**

Venous blood (2ml) will be collected from all cases twice daily for a minimum of 5 days after starting treatment. This 2ml sample is sufficient to evaluate viral and haematological parameters. At the time of discharge, a 5ml blood sample will be collected to measure dengue-specific T cell responses.

**Vomiting protocol**

In the event the patient vomits within 5 minutes of receiving the study drug, then the patient will be re-dosed.

# **Analysis plan- primary endpoints**

Baseline variables at randomization will be compared between the 2 treatment groups and analysed using the Kruskal-Wallis test for continuous variables and the chi-square test (or Fisher’s exact test) for categorical variables. Only study subjects who have detectable NS1 antigenaemia or viraemia (detected by PCR) at the time of study entry will be analysed for the primary endpoint measures. The primary endpoints are time to negative DENV NS1 antigenaemia in plasma and time to negative reverse transcriptase (RT)-PCR detection of viral RNA in plasma. Comparison of Kaplan-Meier survival curves (representing proportion of cases NS1 or PCR positive) between treatment groups using log-rank test will be performed. Adjusted treatment effect will be estimated and independent predictors will be identified using Cox regression analysis with stepwise forward variable selection procedure.

# **Analysis plan- secondary endpoints**

Analysis of secondary outcome measures will be performed at the end of the trial. Secondary endpoints include;

1. Fever clearance time (Time to first fall ≤ 37.5^0^C, axillary, and to remain ≤ 37.5^0^C for 48 hours)
2. Time to plasma concentrations of the pro-inflammatory cytokines TNF, IL-6, IL-1B, IL12, IL-8 and the anti-inflammatory, IL-10 to return to normal range (defined a priori)
3. Time to SGOT, SGPT to return to normal range (defined a priori)
4. Time to platelet count to exceed >100,000 per ul

These secondary outcome measures will be analysed by Kaplan-Meier estimates with comparison of curves between treatment groups using log-rank test. Adjusted treatment effect will be estimated and independent predictors will be identified using Cox regression analysis with stepwise forward variable selection procedure.

**Other secondary endpoints include;**

1. Frequency of grade 3 and 4 adverse events as defined in Appendix 1 that are probably or definitely related to the intervention will be compared between treatment groups.
2. Frequency of new, clinically significant complications after commencement of treatment, including;
   1. Dengue shock syndrome as defined by WHO criteria
   2. Gastrointestinal bleeding requiring transfusion with blood or blood products
3. ELISPOT assays to measure DENV-specific T cell responses. The magnitude and breadth of DENV-specific T cell responses will be compared between treatment groups.
4. The magnitude of gene expression changes in peripheral blood measured by microarray or RT-PCR will be compared between treatment groups.

Frequencies of these secondary endpoints will be compared between treatment groups**.** Statistical analysis will be by parametric (student t-test) or non-parametric (Mann-Whitney U test) approaches. Comparison of proportions will be performed using chi-square (or Fishers exact) test.

**Subgroup analysis**

The following sub-group analyses will be performed:

1. Comparison of primary and secondary outcomes for those with primary or secondary dengue
2. Comparison of primary and secondary outcomes for each DENV serotype
3. Comparison of primary outcome for cases with DENV viraemias >10,000 cDNA equivalents per ml at the commencement of treatment
4. Comparison of primary outcome for cases with NS1 antigenaemias >500ng/ml at the commencement of treatment
5. In subjects with at least 4 consecutive positive RT-PCR, we will determine the slope of the line representing changes in viral load over time.

**Hospital costs**

For patients in the study, the OUCRU will pay hospital costs that are consistent with routine care of uncomplicated dengue.

###### References

1. Modis, Y., S. Ogata, D. Clements, and S. C. Harrison. 2004. Structure of the dengue virus envelope protein after membrane fusion. *Nature 427:313*.

2. Ros, C., C. J. Burckhardt, and C. Kempf. 2002. Cytoplasmic trafficking of minute virus of mice: low-pH requirement, routing to late endosomes, and proteasome interaction. *J Virol 76:12634*.

3. Diaz-Griffero, F., S. A. Hoschander, and J. Brojatsch. 2002. Endocytosis is a critical step in entry of subgroup B avian leukosis viruses. *J Virol 76:12866*.

4. Randolph, V. B., and V. Stollar. 1990. Low pH-induced cell fusion in flavivirus-infected Aedes albopictus cell cultures. *J Gen Virol 71 (Pt 8):1845*.

5. Ducharme, J., and R. Farinotti. 1996. Clinical pharmacokinetics and metabolism of chloroquine. Focus on recent advancements. *Clin Pharmacokinet 31:257*.

6. McChesney, E. W., W. F. Banks, Jr., and R. J. Fabian. 1967. Tissue distribution of chloroquine, hydroxychloroquine, and desethylchloroquine in the rat. *Toxicol Appl Pharmacol 10:501*.

7. Mackenzie, A. H. 1983. Pharmacologic actions of 4-aminoquinoline compounds. *Am J Med 75:5*.

**Figure 1. Activity of chloroquine against Dengue Virus (NGC) in CFI assay**

CFI assay:

-1

1 da

2 daysddays

0

Cell seeding

1. Virus infection;

1. ELISA to detect viral E protein production;

2. Fluorescent endpoint assay to measure cytotoxicity of testing compounds

(day)

Day-1. Seeding of 20,000 cells into 96-well microtiter plates

Day 0. Infection of cells with NGC at MOI=0.3 in the presence of chloroquine (0.039 ~ 20uM in 2X series dilution);

Day 2. Virus load is measured by quantifying the amount of E protein produced using ELISA. IC50s are calculated by GraphPad Prism V.3.02. In addition, the amount of viable cells are quantified using fluorescent endpoint assay(propidium iodine staining).

**Figure 2. KPM survival analysis of a pilot study of chloroquine use in dengue patients. Shown is the proportion of adult patients (n=20) with NS1 antigenaemia since beginning randomised treatment (chloroquine or placebo).**
